# Insulin-Producing Cells in the *Drosophila* Brain also Express Satiety-Inducing Cholecystokinin-Like Peptide, Drosulfakinin

**DOI:** 10.3389/fendo.2012.00109

**Published:** 2012-08-31

**Authors:** Jeannette A. E. Söderberg, Mikael A. Carlsson, Dick R. Nässel

**Affiliations:** ^1^Department of Zoology, Stockholm UniversityStockholm, Sweden

**Keywords:** peptide hormones, insulin signaling, satiety regulation, feeding behavior, food choice, *Drosophila melanogaster*

## Abstract

Regulation of meal size and assessing the nutritional value of food are two important aspects of feeding behavior. The mechanisms that regulate these two aspects have not been fully elucidated in *Drosophila*. Diminished signaling with insulin-like peptides *Drosophila* insulin-like peptides (DILPs) affects food intake in flies, but it is not clear what signal(s) mediates satiety. Here we investigate the role of DILPs and drosulfakinins (DSKs), cholecystokinin-like peptides, as satiety signals in *Drosophila*. We show that DSKs and DILPs are co-expressed in insulin-producing cells (IPCs) of the brain. Next we analyzed the effects of diminishing DSKs or DILPs employing the Gal4-UAS system by (1) diminishing DSK-levels without directly affecting DILP levels by targeted *Dsk*-RNAi, either in all DSK-producing cells (DPCs) or only in the IPCs or (2) expressing a hyperpolarizing potassium channel to inactivate either all the DPCs or only the IPCs, affecting release of both peptides. The transgenic flies were assayed for feeding and food choice, resistance to starvation, and for levels of *Dilp* and *Dsk* transcripts in brains of fed and starved animals. Diminishment of DSK in the IPCs alone is sufficient to cause defective regulation of food intake and food choice, indicating that DSK functions as a hormonal satiety signal in *Drosophila*. Quantification of *Dsk* and *Dilp* transcript levels reveals that knockdown of either peptide type affects the transcript levels of the other, suggesting a possible feedback regulation between the two signaling pathways. In summary, DSK and DILPs released from the IPCs regulate feeding, food choice and metabolic homeostasis in *Drosophila* in a coordinated fashion.

## Introduction

Caloric intake is tightly regulated in animals to ensure energy stores sufficient for daily activity, and, at least in mammals, the nutrient consumption is also controlled to maintain body weight near constancy over extended periods (Woods et al., 2000; Murphy and Bloom, 2006; Murphy et al., 2006; Woods and D’Alessio, 2008; Al-Anzi et al., 2010). Since caloric intake is known to affect body weight, insulin signaling, and healthy life span (Kenyon et al., 1993; McMinn et al., 2000; Tatar et al., 2001, 2003; Broughton et al., 2005; Partridge et al., 2005; Giannakou and Partridge, 2007; Ja et al., 2009; Moran and Dailey, 2009), it is of interest to understand regulation of feeding. In most animals food intake depends on meal size and frequency of meals (Murphy and Bloom, 2006; Woods and D’Alessio, 2008; Al-Anzi et al., 2010; Cognigni et al., 2011), but what mechanisms control initiation, maintenance, and termination of feeding, and how is food quality assessed in relation to hunger? Due to problems in modern society with obesity, diabetes, and other metabolic disorders, large efforts have been made, especially in mammals, to investigate the roles of neuropeptides and peptide hormones in regulation of feeding and metabolism (McMinn et al., 2000; Strader and Woods, 2005; Sandoval et al., 2008; Moran and Dailey, 2009; Barth, 2011).

Although insects have been used quite extensively for analysis of feeding behavior, since the seminal work on the blowfly *Phormia regina* (Dethier, 1976), they have only recently been adopted for studies of peptidergic regulation of food choice, food intake, and metabolism. Thus far there is evidence for several peptides acting at different levels in regulation of feeding in insects: leucokinins (Al-Anzi et al., 2010), neuropeptide F (NPF; Wu et al., 2003, 2005a), short neuropeptide F (sNPF; Lee et al., 2004, 2008), insulin-like peptides (Broughton et al., 2010; Cognigni et al., 2011), *Hugin* derived peptides (Melcher and Pankratz, 2005), sulfakinins (Wei et al., 2000; Maestro et al., 2001; Wu et al., 2003; Downer et al., 2007; Meyering-Vos and Muller, 2007), allatostatins (Aguilar et al., 2003; Hergarden et al., 2012), and sex peptide (Carvalho et al., 2006). It is, however, not yet clear how these peptide signals are coordinated to control the initiation, maintenance, and termination of feeding (see Audsley and Weaver, 2009; Nässel and Winther, 2010). In mammals peptidergic signaling in regulation of feeding and satiety is very complex and involves among others cholecystokinin (CCK), neuropeptide Y, peptide YY, pancreatic polypeptide, leptin, ghrelin, and insulin (Strader and Woods, 2005; Wynne et al., 2005; Murphy et al., 2006; Sandoval et al., 2008; Woods and D’Alessio, 2008; Barth, 2011; Dagher, 2012). Here we investigate the coordinated roles in feeding in *Drosophila* of the CCK-like *Drosophila* sulfakinins (drosulfakinins, DSKs), and *Drosophila* insulin-like peptides (DILPs).

The reason for targeting these peptides is the observation that a set of median neurosecretory cells (MNCs) in the pars intercerebralis of larval brain of *Drosophila* express both DSKs and DILPs (Park et al., 2008) and thus may act in a coordinated fashion in regulation of feeding and metabolism. Also, it is of interest to determine the specific role of DSKs in these MNCs since several studies have analyzed the effect of genetically ablating or silencing these cells (see; Broughton et al., 2005; Broughton et al., 2010; Cognigni et al., 2011), and the phenotype obtained after this ablation is likely to be a result of diminishing both types of peptides. In other words, do these MNCs serve functions additional to the known DILP-mediated ones?

In *Drosophila* and other animals insulin signaling has been implicated in regulation of growth, fecundity, metabolic homeostasis, stress resistance, and longevity (Brogiolo et al., 2001; Wu and Brown, 2006; Baker and Thummel, 2007; Giannakou and Partridge, 2007; Fontana et al., 2010; de la Rosa and de Pablo, 2011; Antonova et al., 2012). Eight genes encoding DILPs are known in *Drosophila* and are differentially expressed in a stage- and tissue-specific manner (Brogiolo et al., 2001; Grönke et al., 2010; Colombani et al., 2012; Garelli et al., 2012). Three of these, encoding DILP2, 3, and 5, are co-expressed in a set of MNCs of the brain designated insulin-producing cells, IPCs (Cao and Brown, 2001; Ikeya et al., 2002; Rulifson et al., 2002; Karpac et al., 2009). These three *Dilp* genes are under individual transcriptional control, depending on various nutritional, or stress conditions, or genetic manipulations of IPC function (see, e.g., Ikeya et al., 2002; Géminard et al., 2009; Karpac et al., 2009; Broughton et al., 2010; Grönke et al., 2010; Slack et al., 2010; Birse et al., 2011). This is a feature that may indicate that the three DILPs have different functions. On the other hand, the DILPs display a certain degree of functional redundancy and the transcriptional levels of other DILPs increase if one is knocked down (Broughton et al., 2008; Grönke et al., 2010).

Partial genetical ablation of the IPCs in the brain in late larval stages, prolongs lifespan, reduces fecundity, changes both lipid, and carbohydrate metabolism and increases the resistance to both starvation and oxidative stress (Broughton et al., 2005). Ablation of the IPCs in the early larval stages leads to developmental delay, growth retardation, and increased circulating carbohydrate levels (Partridge and Gems, 2002; Broughton et al., 2005). Flies with deleted or inactivated IPCs also display altered feeding behavior (Broughton et al., 2010; Cognigni et al., 2011). The question is to what extent this effect on feeding is due to DILPs or the co-localized DSKs, since ablation or inactivation of the IPCs affect both types of peptides and sulfakinins have been shown to induce satiety (Wei et al., 2000; Maestro et al., 2001; Wu et al., 2003; Downer et al., 2007; Meyering-Vos and Muller, 2007).

Drosulfakinins and sulfakinins of other insects are related to members of the vertebrate CCK peptide family, based on similarities in amino acid sequence and the requirement of a sulfated tyrosyl residue for bioactivity (Nachman et al., 1986a,b, 1988; Nichols et al., 1988; Staljanssens et al., 2011). Also the DSK receptors (CG6857 and CG6881) are considered related to the gastrin/CCK receptors, based on similarities in amino acid sequence and gene organization (Kubiak et al., 2002; Hauser et al., 2006; Janssen et al., 2008; Staljanssens et al., 2011). Like CCK in mammals (Moran et al., 1998; Dockray, 2009; Moran and Dailey, 2009), sulfakinins are satiety-inducing signals in locusts, crickets, cockroaches, and flies (see Maestro et al., 2001; Wu et al., 2003; Downer et al., 2007; Meyering-Vos and Muller, 2007; Wicher et al., 2007; Nichols et al., 2008). The two peptides (DSK-1 and 2) of the *D. melanogaster* sulfakinin gene (*Dsk*) have also been shown to play roles in locomotion and odor preferences and a third peptide DSK-0 on the same gene induces crop contractions (Nichols et al., 1988; Duttlinger et al., 2002; Palmer et al., 2007). It should be noted that *Dsk* and its peptide products are expressed by several other neurons in the *Drosophila* brain in addition to the IPCs (Nichols, 1992; Nichols and Lim, 1996; Park et al., 2008). However, the IPCs seem to be the only DSK-producing neurons that are neurosecretory with axons terminating in neurohemal areas of the corpora cardiaca, aorta, and anterior intestine (see Nichols and Lim, 1996; Park et al., 2008, unpublished observations). Here, we investigated the functional role of DSKs in the IPCs in comparison with global knockdown of all DSK in the brain. We utilized different sets of Gal4- and UAS-lines in crosses, to elucidate the role of DSKs in relation to DILPs in the IPCs. To manipulate DSK-levels without affecting DILP levels, we utilized a *Dsk*-RNAi construct, either in all DSK-producing cells (DPCs; *Dsk*-Gal4) or only the IPCs (*Dilp2*-Gal4). We also inactivated the IPCs or DPCs with a hyperpolarizing potassium channel. These experiments separate the global role of DSKs in all DPCs and the role of DSKs co-localized with DILPs in IPCs. We tested the transgenic flies in various assays of feeding, food choice, starvation, and for levels of *Dilp* and *Dsk* transcripts in fed and starved flies. Our data suggest that DSKs in IPCs are sufficient for regulation of satiety in larvae and adult flies.

## Materials and Methods

### Fly strains and husbandry

All flies were reared at 25°C on a standard yeast/corn meal/agar medium, under 12:12 h light:dark conditions. The following fly lines were used in the experiments: *Dsk*-Gal4 (Park et al., 2008) donated by Jae H. Park, Knoxville, USA (Park et al., 2008) and *Dilp2*-Gal4 (Ikeya et al., 2002) originally from Ernst Hafen, Zürich, Switzerland (donated by S. Broughton, Lancaster, UK). These strains were used to drive expression of target genes in the DPCs and the IPCs, respectively, by means of the binary Gal4-UAS (Upstream Activating Sequence) system. To diminish the expression of DSKs, we used a UAS-*Dsk*-RNAi line (CG18090; Transformant ID14201) obtained from the Vienna *Drosophila* RNAi Center (VDRC). A UAS-*Dilp5*-RNAi line (CG32273; Transformant ID 49520) was obtained from VDRC. These transformants have no recorded off targets. Four to six days old adult progeny of the crosses between Gal4 and UAS lines were collected for experiments, and as controls we used these parental strains crossed to *w^1118^* flies [from Bloomington *Drosophila* Stock Centre (BDSC) at Indiana University, Bloomington, IN, USA]. A *Dilp2,3,5* mutant (Grönke et al., 2010) was provided by S. Grönke and L. Partridge (London, UK). These *Dilp* deletions were generated by ends out homologous recombination and shown to be null alleles (Grönke et al., 2010). As wild type controls we used the *w^1118^* strain. To inactivate the DPCs or IPCs through membrane hyperpolarization, we employed UAS-*dOrk1*, a construct with a constitutively active K^+^-channel (Nitabach et al., 2002), obtained from BDSC. In all experiments on adults, only male flies were used.

### Electrical silencing of DPCs and IPCs

A means to silence neuronal signaling is to suppress electrical activity in the membrane of the neuron. This can be achieved by expressing potassium channels that remain open even at resting membrane potential, allowing for an increased flux of potassium ions out of the neuron that hyperpolarizes it and prevents it from signaling (Nitabach et al., 2002). We utilized a UAS-*dOrk1*-line (Nitabach et al., 2002) to express a constitutively activated potassium channel and inactivate either IPCs or DPCs. This method enabled us to silence the cells and affect both DSK and DILPs in the IPCs without ablating the cell, thus avoiding the compensatory developmental mechanisms that might occur when the cells undergo apoptosis.

### Immunocytochemistry and GFP expression

Drosulfakinin localization in the brain was detected using an antiserum against DSK donated by Ruthann Nichols (Ann Arbor, MA, USA). A anti-rabbit antiserum to DILP2 (Cao and Brown, 2001) provided by Mark Brown (Athens, GA, USA) was used to visualize the IPCs. UAS-mCD8-GFP flies crossed with Gal4 drivers to visualize GFP expression in cells in order to perform double-labeling with peptide antisera. A mouse monoclonal antibody to GFP (#A-11120, Molecular Probes, Invitrogen) or a rat monoclonal antibody to mCD8 (#MCD0800; Molecular Probes, Invitrogen) were used to amplify the fluorescence intensity of the mCD8-GFP signal.

Fly brains were fixed in 4% paraformaldehyde (PFA) in 0.1 M sodium phosphate buffer (PB; pH 7.4). The dissected brains were incubated with primary antibody diluted in 0.01 M phosphate-buffered saline (PBS; pH 7.4), with 0.25% Triton-X, and 0.5% bovine serum albumin (BSA) for 48–72 h. A thorough washing in PBS containing 0.25% Triton-X (PBS-Tx) was followed by incubation in secondary antibody: Alexa 546-conjugated goat anti-rabbit antiserum (#A-11010, Invitrogen) at 1:1500. Specimens were imaged with a Zeiss LSM 510 confocal microscope (Jena, Germany) and processed with Zeiss LSM software and edited for contrast and brightness in Adobe Photoshop CS3 version 10.0.1.

### Food intake assays

Different assays are available for measuring food consumption in *Drosophila*. We slightly modified the protocol of Al-Anzi et al. (2010). Flies were starved for 18 h on 0.5% aqueous agarose. Thereafter, they were allowed to feed on standard food for 15 min, and were transferred onto 1% indigo (Sigma-Aldrich, #229296) colored food for 15 min. The flies were observed under a light microscope and scored for crop and gut color. Flies that consumed indigo blue food had blue abdomens, whereas those that did not feed on the blue food after the transfer had white abdomens. Preliminary experiments were conducted where the flies’ first meal was dyed with 1% carmine red (Sigma-Aldrich), to visualize that the flies fed on the first meal. For the final scoring however, white and indigo blue food was used in order to more accurately score the flies. In all experiments on adult *Drosophila* only male flies were utilized and we report on the amount of blue dye ingested.

### Absorbance measure for quantification of ingested food

To quantify the food intake of the flies more accurately; the absorbance of the ingested dye was measured as previously described (Edgecomb et al., 1994; Meunier et al., 2007). Flies in groups of 20 were starved for 1 day on 0.5% agarose. The flies were transferred into vials containing 1% sucrose in 1% agarose. After 20 min, the flies were again transferred into new vials containing 1% sucrose in 1% agarose, but with 1% indigo dye (Sigma-Aldrich), and left to feed for another 15 min. The tested flies were homogenized with a mortar in PBS and centrifuged for 3 min. The supernatant was treated with *n*-heptane to remove lipid debris and the absorbance of the dye was measured in a Jenyway Genova spectrophotometer at 620 nm.

### Larval food choice test

Feeding third instar larvae (96-h-old) were collected and placed in Petri dishes, 15 cm in diameter. These Petri dishes had previously been prepared with an inner circle of sugar-free medium or medium with 2% caffeine, whereas the outer circle was made from standard food. The larvae were allowed to feed at libitum for 15 min and thereafter the larvae present in the two different circles were counted.

### Adult food choice test

The adult food choice test was adapted from Ribeiro and Dickson (2010) and Al-Anzi et al. (2010). A 96-well plate was filled alternately in a checkerboard pattern with food with our without caffeine (or without sugar in separate tests). The caffeine-spiked (or sugar-free) food was dyed with blue dye. The sedated flies were placed in the blue non-desirable food but were allowed to walk out of the well to choose another food well (they could not fly due to a cover). After feeding, the flies’ abdomens were visually inspected under a microscope and scored for gut coloring. The assay was performed with the caffeine-spiked (or sugar-free) food being either dyed or undyed in order to eliminate possible effects of the dye (i.e., reversing the color coding of the food). In each test we used a minimum of 300 flies of each genotype. A test was repeated four times with 60–110 flies in each replicate.

### Starvation assay

To investigate the role of DSK and DILPs in starvation resistance, flies were subjected to starvation according to the protocol of Lee and Park (2004). Male flies, aged 4–8 days, were anesthetized using CO_2_ and placed individually in 2 ml glass vials containing 0.5% aqueous agarose kept at 25°C. This provided them with water but no food. Dead flies were counted every 12 h and the resulting survival curve was analyzed with Log-rank test (Mantel–Cox) in Prism GraphPad 5.0.

### RNA extractions and quantitative real time PCR

Total RNA from whole heads was extracted by using TRIzol (GIBCO) according to the manufacturer’s protocol. The RNA was treated with DNase to remove any residual genomic DNA (Turbo DNA-freeTM, Ambion). Treated mRNA was reverse transcribed to cDNA using Quantitect Reverse Transcription Kit (Qiagen) according to the manufacturer’s instructions. The *dilp* primers were as follows: *dilp*2F (forward), TCTGCAGTGAAAAGCTCAACGA; *dilp*2R (reverse), TCGGCACCGGGCATG; *dilp*3F, AGAGAACTTTGGACCCCGTGAA; *dilp*3R, TGAACCGAACTATCACTCAACAGTCT; *dilp*5F, GAGGCACCTTGGGCCTATTC; and *dilp*5R, CATGTGGTGAGATTCGGAGCTA. The *dsk* primers used were: *dsk* (forward) CCGATCCCAGCGCAGACGAC and *dsk* (reverse) TGGCACTCTGCGACCGAAGC.

PCR was performed using Taqman Universal PCR Master Mix according to the manufacturer’s instructions (Applied Biosystems), with the exception that 25 μl reaction volumes were used, on an ABI Prism 7000 (Applied Biosystems). Endogenous genetic control (*rp49*) primers were as follows: *rp49*F, CACACCAAATCTTACAAAATGTGTGA; and *rp49*R, AATCCGGCCTTGCACATG.

All samples were analyzed in triplicates, and the measured concentration of mRNA was normalized relative to endogenous rp49 control values. Experiments were made in three replicates starting from new RNA extraction. The relative levels of a given mRNA were quantified from the normalized data according to the ΔCt analysis (Livak and Schmittgen, 2001).

### Statistics

All experiments were run at least in triplicate with a minimum of 40 flies of each genotype in each replicate. Statistics were performed using GraphPad Prism 5.0. The starvation and oxidative stress survival results were analyzed with Log-rank test (Mantel–Cox) and the food intake assay results were analyzed with one-way ANOVA and Tukey *post hoc* test. The quantitative real time PCR (qPCR) results were analyzed with one-way and two-way ANOVA.

## Results

### DSK and DILPs co-localize in the insulin-producing cells

A subset of the median secretory cells of the protocerebrum produces DILP2, 3, and 5. These cells, designated IPCs have axon terminations in the corpora cardiaca, the aorta, the crop, and the anterior midgut, all presumed to be neurohemal release sites for circulating hormones (Cao and Brown, 2001; Rulifson et al., 2002; Cognigni et al., 2011). The presumed dendrites of the IPCs are located in the pars intercerebralis, dorsally in the protocerebrum.

**Figure 1:**
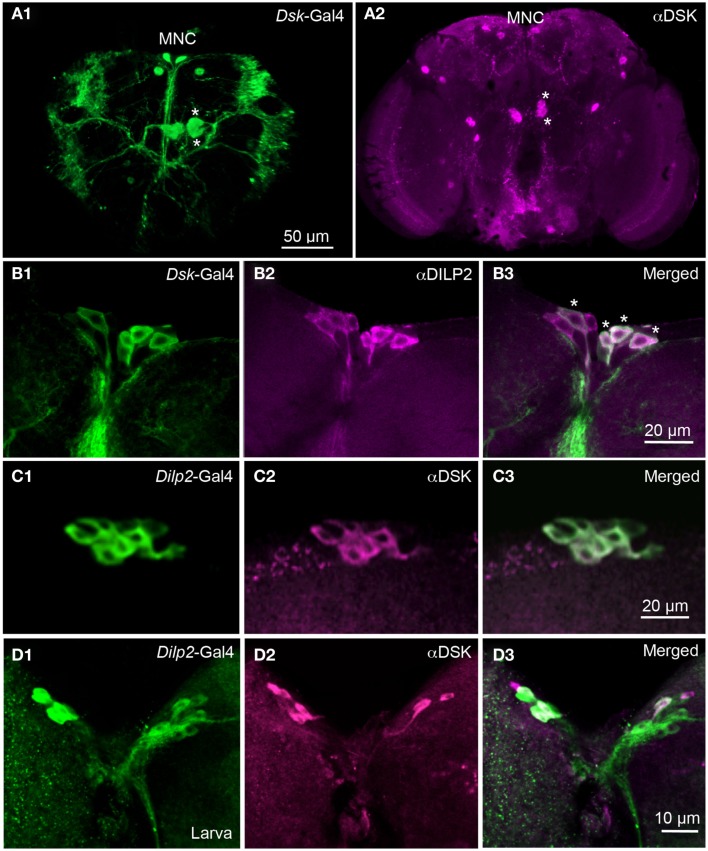
***Dilp2*-expressing IPCs also display DSK immunoreactivity**. **(A1)** *Dsk*-Gal4 driven GFP reveals expression in median neurosecretory cells (MNC) in the pars intercerebralis of the adult brain. Two additional larger neurons can be noted in the superior protocerebrum. Four large cells, two in each hemisphere (asterisks) of the midbrain extend arborizations toward the lateral horn, and the lateral protocerebrum. **(A2)** Labeling with DSK antiserum in the adult brain reveals immunoreactivity in the MNCs and in other cells in the protocerebrum. Two cells marked with asterisks correspond to the cells marked in **(A1)**. Arborizations can be seen in the pars intercerebralis and in the subesophageal ganglion and descending to the ventral nerve cord **(B1–B3)**. The adult IPCs co-localize *Dsk*-Gal4 expression (green) and DILP2 immunoreactivity (magenta). Cells with co-localized markers are indicated by asterisks **(C1–C3)**. Co-localization of *Dilp2*-Gal4-driven GFP and DSK-immunolabel in IPCs of the adult fly. The majority of the GFP expressing IPCs also display DSK immunoreactivity **(D1–D3)**. In the larval brain the IPCs are double-labeled with antiserum to DSK and *Dilp2*-Gal4-driven GFP. The markers co-localize in the majority of the IPCs, but with stronger DSK labeling in some cells. In these images the intensity of mCD8-GFP fluorescence was improved by using antiserum to GFP or CD8.

To visualize neuronal *Dsk* expression we used a *Dsk*-Gal4 line (Park et al., 2008) to drive UAS-mCD8-GFP. We found *Dsk*-Gal4 expression in several neuron groups in the adult brain, including cells resembling the IPCs (Figures 1A1,B1). Using anti-DSK antiserum, we labeled similar neurons in the brain (Figures 1A2,B2). In addition, we found DSK-immunolabeling and Gal4 expression in interneurons in other parts of the protocerebrum and subesophageal ganglion (Figure 1A2), also shown by Nichols et al. (Nichols and Lim, 1996; Nichols et al., 1997a,b). Since we found DSK immunoreactivity and *Dsk*-Gal4 expression in several cells among the median secretory cells, probably including the IPCs, we applied DILP2 antiserum to adult brains for identification. Thus, we confirmed that the *Dsk*-GFP-expressing median secretory cells also are DILP immunopositive (Figures 1B1–B3) and conversely we showed that most of the *Dilp2*-Gal4-expressing IPCs react with antiserum to DSK (Figures 1C1–C3). Also in the third instar larvae many of the IPCs co-express DSK immunoreactivity (Figures 1D1–D3) as previously shown (Park et al., 2008). Not all IPCs express DSK in flies or larvae and the DSK expression is somewhat variable.

Since most of the IPCs in the adult brain also express DSKs, it is suggestive that these cells signal with both DILPs and DSKs. Since the IPCs are neurosecretory cells we expect that the DSKs are released from these cells as circulating hormones. Hence, we went on to investigate the functional role of DSKs in the IPCs.

### Food intake is regulated by DSKs in the IPCs

The co-localization of DILPs and DSKs in the IPCs suggests that the hormonal actions of the two sets of peptides may be functionally coordinated. DSKs have previously been proposed to be satiety signals in blowflies and other insects (Maestro et al., 2001; Wu et al., 2003; Downer et al., 2007; Meyering-Vos and Muller, 2007; Nichols et al., 2008) and silencing of IPCs has been shown to affect feeding in *Drosophila* (Broughton et al., 2010; Cognigni et al., 2011). We set out to determine the function of the DSK signaling from the IPCs as compared to signaling from the entire population of DPCs. More specifically we ask whether the DSKs produced in the IPCs are sufficient to regulate feeding and satiety? In all experiments on adult *Drosophila* only male flies were utilized.

In the following experiments flies were starved for 18 h ahead of the experiment and were subsequently placed on standard food for 15 min before transferred to indigo colored food. This duration was chosen since a normal meal lasts about 15 min (Al-Anzi et al., 2010).

**Figure 2:**
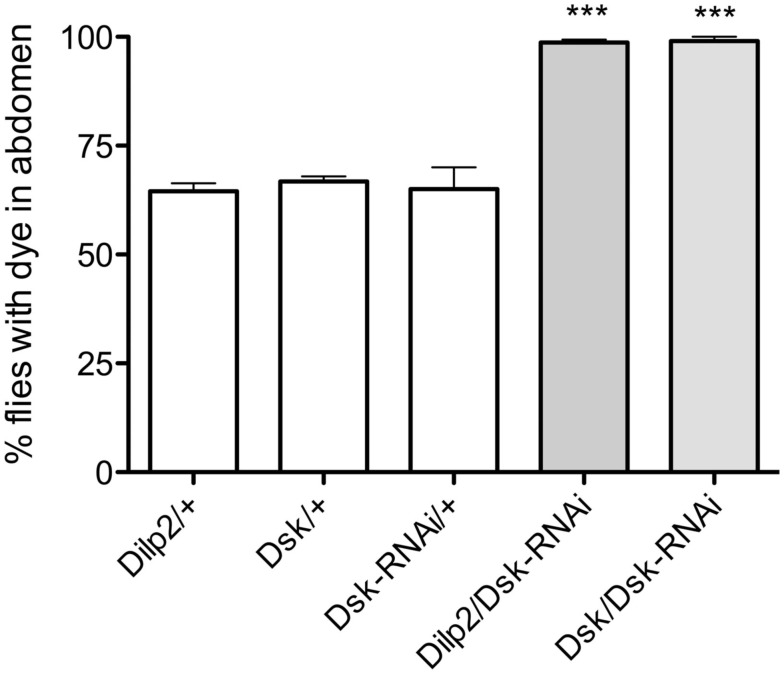
**Drosulfakinin deficient flies consume more food than controls**. Ingested indigo dyed food was visually detected in the abdomen of transgene flies. In all experiments in this and other figures parental controls were crossed to wildtype *w^1118^* flies (reported as, e.g., Dilp2/+). After 15 min of feeding, an average of 98.6% of the flies with Dsk-knockdown in all DSK-expressing cells, DPCs (Dsk/Dsk-RNAi) and 99% of the flies with Dsk-RNAi in IPCs (Dilp2/Dsk-RNAi) had fed on blue food, compared to only 64.5 and 66.7% of respective controls, Dilp2/+, Dsk/+, and Dsk-RNAi/+ (*p* < 0.001, *n* = 90–112 for the different genotypes, One-way ANOVA). All experiments were performed in three replicates, ***p < 0.001.

To investigate effect of DSK-knockdown in feeding, *Dsk*-RNAi was driven both in IPCs (with *Dilp2*-Gal4) and in DPCs (with *Dsk*-Gal4). Driving *Dsk*-RNAi in either cell population rendered the same phenotype: nearly 100% of the flies with diminished *Dsk*-levels continued to feed on the indigo colored food after transfer, whereas only approximately 70% of the controls did (*p* < 0.001 for both genotypes, One-way ANOVA; Figure 2). Control flies apparently became satiated from the first meal and did not feed on the blue food to the same extent as the test flies. The *Dsk* deficient flies however, displayed a defect in regulation of food intake as they continued to feed on the blue food. These experiments show that *Dsk*-RNAi in the IPCs is sufficient to induce a defective feeding phenotype and no additional effect was detected after knocking down *Dsk* in all of the DPCs.

**Figure 3:**
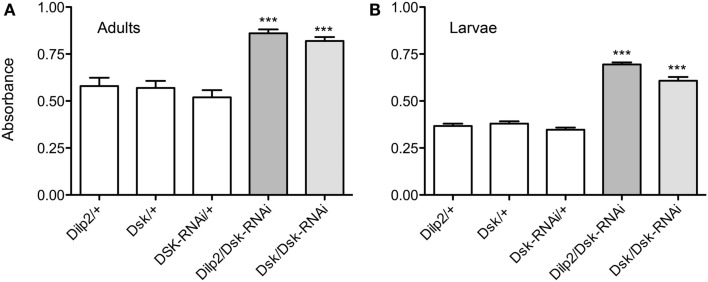
**Diminishing Dsk causes adult flies and larvae to ingest more food**. For quantification of food intake the absorbance of the ingested food dye in flies and larvae was measured at 625 nm by spectrophotometry. The absorbance of ingested colored food was measured in 20 adult flies or larvae of each genotype, and the experiments were repeated four times (***p < 0.001). **(A)** The absorbance of ingested colored food was measured in adult flies after 15 min of feeding. The mean absorbance was 43% higher in Dsk-Gal4/Dsk-RNAi flies and 43% higher in Dilp2/Dsk-RNAi flies than in respective controls, Dilp2/+, Dsk/+, and Dsk-RNAi/+ (*p* < 0.001, One-way ANOVA), indicating a larger food intake amongst the flies with DSK-knockdown both in all DPCs and only in IPCs. **(B)** DSK regulates food intake also in larvae. After 15 min of feeding, a mean absorbance of 0.60 in Dsk-Gal4/Dsk-RNAi and 0.695 in Dilp2/Dsk-RNAi larvae was measured compared with 0.35, 0.37, and 0.38 in controls (*p* < 0.001, One-way ANOVA). The Dsk/Dsk-RNAi and Dilp2/Dsk-RNAi larvae displayed an almost 50% higher absorbance than controls. For all experiments manipulations the IPCs alone were sufficient to induce a feeding phenotype.

For a more precise quantification of the food intake we measured the absorbance of the ingested dye (Meunier et al., 2007). We detected a significantly higher absorbance in the *Dilp2-Gal4*/*Dsk*-RNAi and *Dsk-*Gal4-*Dsk-*RNAi flies than in controls (*p* < 0.001 for each genotype, One-way ANOVA; Figure 3A). These results further support that diminishment of DSKs in the IPC is sufficient to cause the observed orexogenic phenotype.

A feeding test was also carried out in larvae to examine the effects of DSK manipulation on feeding behavior in earlier developmental stages. The third instar larvae normally feed continuously and using dyed food enabled us to obtain a measure of the amount of ingested food over time. The *Dsk*-RNAi larvae ingested more food than controls, both when the construct was driven in IPCs and in the DPCs; they displayed approximately 20% higher food dye absorbance (*p* < 0,001, for both genotypes compared to controls, One-way ANOVA; Figure 3B). Again, this suggests that depleting DSKs only from the IPCs is sufficient to cause a deregulation of feeding and cause an increased food intake. Thus, DSKs seem to function as a satiety signal both in larvae and in adults.

### Flies deficient in DSKs display deregulated food choice behavior

Since we could detect a defect in regulation of food intake in DSK deficient flies, we were also interested in whether DSKs play a role in food preference of the flies. Studies have previously linked deregulated food intake with aberrant food choice behavior. Flies that were made deficient in NPF, or its receptor, did not display a normal behavior in the choice between standard or distasteful (noxious) or inaccessible more solid food (Wu et al., 2005a,b; Lingo et al., 2007).

**Figure 4:**
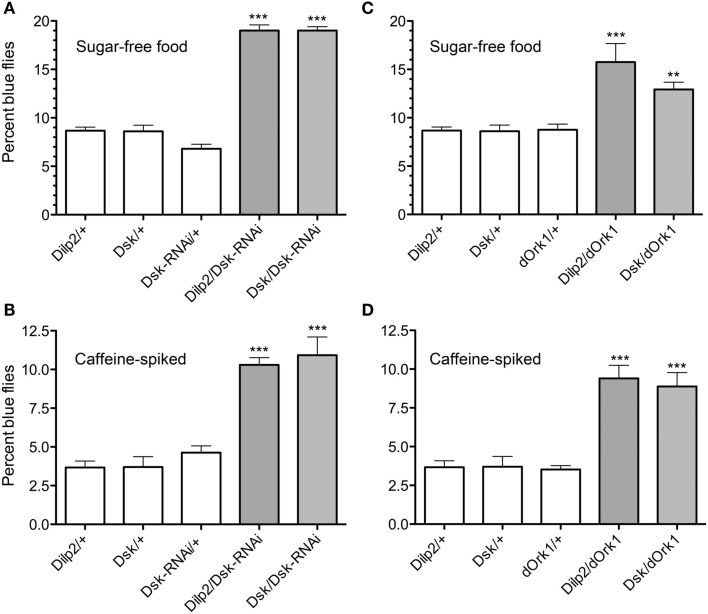
**Drosulfakinin affects food quality discrimination**. Ten adult male flies of each genotype were used in each test, and the test was repeated five times (**p < 0.01, ***p < 0.001, One-way ANOVA, Tukey post test). Adult male flies were allowed to choose between caffeine-spiked, sugar-free, and standard fly food as described in “Materials and Methods.” The standard food was white and the two non-preferred foods with blue dye. Flies were put in wells with non-preferred food and allowed choice to feed or move to an adjacent well with standard food. Between 60 and 110 flies of each genotype were used in each test and each test was repeated four times (**p < 0.01, ***p < 0.001, One-way ANOVA, Tukey post test). Each set of experiments was also run with the coloring of the food reversed (i.e., blue standard food) with similar results (not shown). This figure shows only results with blue non-preferred food; percent flies with blue gut contents were scored. **(A)** Test of sugar-free food. Flies depleted of DSK by the means of *Dsk*-RNAi in IPCs (Dilp2/Dsk-RNAi) or in all DPCs (Dsk/Dsk-RNAi) consume sugar-free (blue) food to a greater extent than respective controls (white bars). **(B)** Test of caffeine-spiked food. Depleting flies of DSK in all DPCs (Dsk/Dsk-RNAi) or in IPCs only (Dilp2/Dsk-RNAi) caused flies to increase their ingestion of caffeine-spiked (blue) food. **(C)** Electrical inactivation of IPCs signaling in adult flies with dOrk1 (*Dilp2*/*dOrk1*) is sufficient to increase the flies’ intake of sugar-free food. Electrical inactivation of all DPCs (Dsk/dOrk1), also significantly increased this food consumption. **(D)** Electrical inactivation of IPCs (*Dilp2*/*dOrk1)* and DPCs (*Dsk*/*dOrk1)* also results in flies that more abundantly feed on caffeine-spiked food.

Adult flies were subjected to a food choice assay where they were exposed both to food that they normally avoid and to standard food (as detailed in material and methods). The distasteful food presented was either bitter due to caffeine, or was sugar-free, thus making it less palatable than the standard food. The non-palatable foods were colored blue and standard food was uncolored. Flies were placed on colored non-palatable food and given the choice to remain feeding on this, or to walk to an adjacent well with standard food. The number of flies that had fed on one or the other of the food types was scored by monitoring ingested color. We observed that flies that expressed *Dsk*-RNAi in either the IPCs or the DPCs were significantly more likely to feed on undesirable food (caffeine-spiked or sugar-free), whereas the control flies fed more on the standard food (*p* < 0.001, One-way ANOVA; Figures 4A,B). Our data suggest that DSK deficient flies remain feeding on non-preferred food, likely as a function of being hungry in absence of a satiety signal, and that *Dsk*-knockdown in IPCs is sufficient for this phenotype.

Next we inactivated neurons of interest with a constitutively active potassium channel, dOrk1 (Nitabach et al., 2002), which hyperpolarizes the neuronal membranes. We expressed dOrk1 in either IPCs or DPCs. This hyperpolarization resulted in the same phenotype as when we diminished *Dsk* through RNAi. The flies consumed more food that was sugar-free or spiked with caffeine than control flies did (Figures 4C,D; *p* < 0,001 for all crosses and food types compared to respective controls, except *p* < 0,01 for *Dilp2*/*dOrk1* in the sugar-free food, One-way ANOVA, Tukey post test).

**Figure 5:**
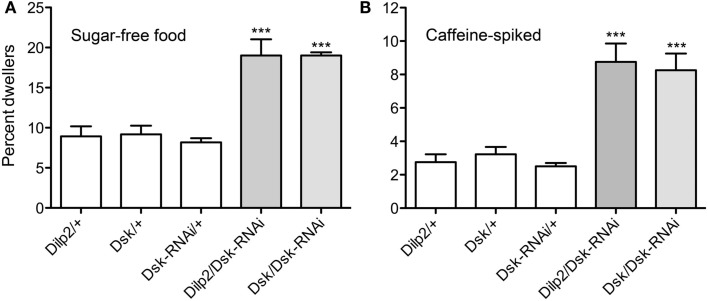
**Drosulfakinins affect larval food choice behavior**. Twenty larvae of each genotype were placed in Petri dishes and allowed to choose between caffeine-spiked food and standard food. The experiment was repeated four times (**p < 0.01, ***p < 0.001, One-Way ANOVA, Tukey post test). **(A)** Test on sugar-free food. *Dsk*-knockdown by RNAi in IPCs (*Dilp2*/*DSK*-RNAi) or DPCs (*Dsk*/*Dsk*-RNAi) resulted in larvae that remained in the sugar-free food to a higher extent than controls (white bars). Results are given as percent dwellers, i.e., larvae remaining in non-preferred food. **(B)** Test of same genotypes on caffeine-spiked food. Again, knockdown of *Dsk* in either IPCs or DPCs resulted in larvae that were more likely to dwell in the caffeine-spiked rather than moving to the standard food.

Furthermore, we examined larval food choice behavior after DSK manipulation. Third instar larvae were given the choice between standard food and sugar-free food or caffeine-spiked food. The larvae were placed on the non-desirable food (of either type) surrounded by standard food and allowed to feed for 15 min. The percentage of larvae that stayed in the non-desirable food rather than moving into the standard food was determined. Control larvae moved away from the caffeine-spiked or sugar-free food and moved into the standard food and about 97% of them were found in this area at the end of the experiment (Figures 5A,B). When *Dsk*-RNAi was driven with *Dilp2*-Gal4 or *Dsk-*Gal4, the larvae stayed in the non-desirable food to a larger extent than control larvae (*p* < 0.001 for both *Dsk/Dsk*-RNAi and *Dilp2*/*Dsk*-RNAi compared to parental controls). It can be remarked that when the larvae were subjected to sugar-free food (Figure 5A), the percentage of control larvae that stayed in that food was larger compared to when they were subjected to caffeine.

In summary, when the flies or larvae have reduced levels of DSK in the IPCs or in the DPCs, or these cells are inactivated, they more readily accept to feed on caffeine-spiked or sugar-free food compared to controls. DSK-knockdown seems to modify the flies’ food preferences, rendering them less choosy, probably as an effect of lack of a satiety signal. Furthermore, DSK signaling from the IPCs seems to be sufficient to mediate this effect.

### Quantification of *Dsk* and *DILP* transcripts after interference with IPCs and DPCs

Since DSK appears to be a hormonal satiety signal that affects feeding behavior in *Drosophila*, it is possible that the levels of *Dsk*/DSK affect the *Dilp*/DILP levels via feedback regulation. We therefore performed qPCR to measure the transcript levels of the different *Dilps* after manipulation of the *Dsk*-levels.

**Figure 6:**
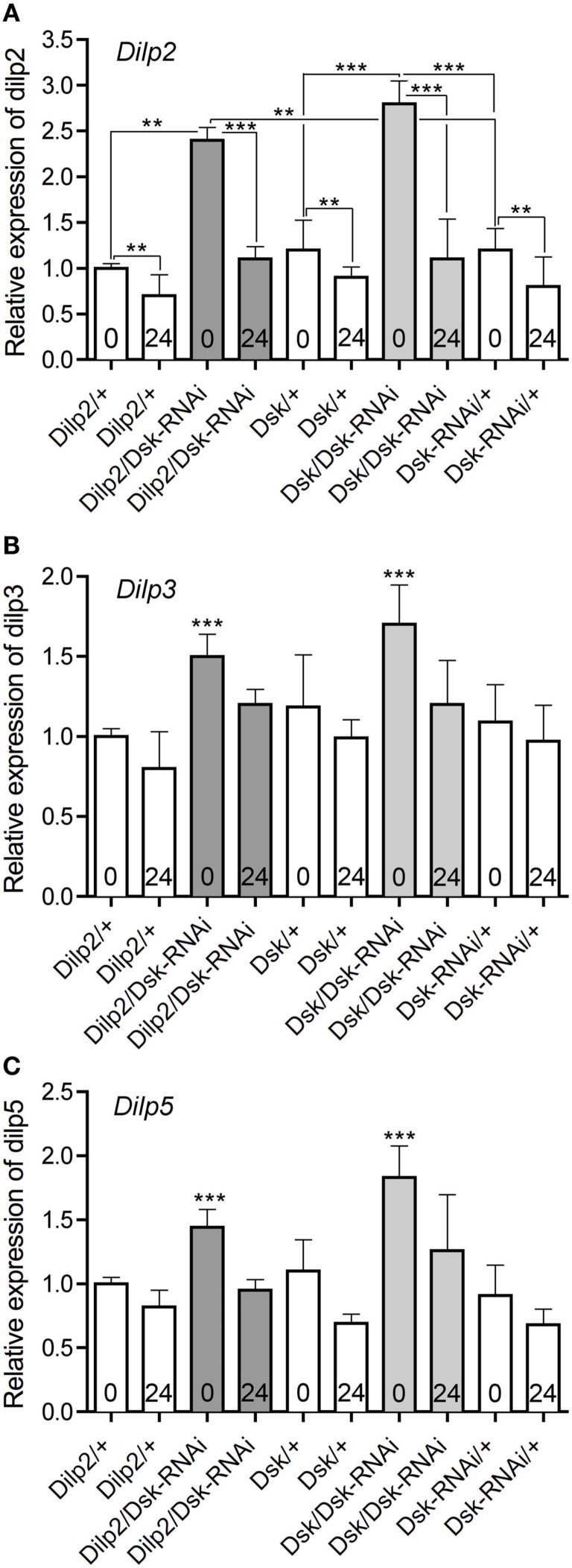
**Relative expression levels of *Dilp* transcripts in *DSK* deficient files**. The effect of *Dsk*-knockdown in the IPCs (*Dilp2*/*Dsk*-RNAi; dark gray bars) and DPCs *Dsk/Dsk*-RNAi (light gray bars) on *Dilp* expression in the brain of adult flies was measured by quantitative real time PCR. Fed flies (bars indicated with 0) and flies starved for 24 h (bars indicated with 24) were monitored. Control flies (Dilp2/+, Dsk/+, and Dsk-RNAi/+) are shown in white bars. Data are shown as mean relative expression ± SD (*n* = 10 for each genotype; each genotype was assayed in triplicate, experiments in triplicate), asterisks denote significant difference compared to controls for fed flies only (**p < 0.01, ***p < 0.001), except in **(A)** where all significance comparisons are shown. Note that the levels of *Dilp2, 3*, and *5* diminish significantly in all genotypes after 24 h starvation (*p* < 0.01, One-way ANOVA, Bonferroni’s *post hoc* test); this is only indicated for *Dilp2* in **(A)**. **(A)** The relative levels of *Dilp2* transcript increase in *Dsk*-knockdown flies compared to parental controls (white bars) in fed flies (*p* < 0.001, One-way ANOVA). Depletion of DSK, either in IPCs or in the DPCs (*Dilp2/Dsk*-RNAi and *Dsk/Dsk*-RNAi) increased *Dilp2* transcripts in fed flies. The levels of *Dilp2* are significantly lower after 24 h starvation for all genotypes (**for all controls and ***for the two sets of *Dsk*-RNAi flies). Note that the *Dilp2* transcript levels in starved (24) experimental flies (*Dilp2/Dsk*-RNAi and *Dsk/Dsk*-RNAi) do not differ significantly from those of starved controls (white bars; n.s., One-way ANOVA), not indicated in figure. **(B)** *Dilp3* transcript levels are higher in fed flies (0) that express *Dsk*-RNAi in IPCs and DPCs (gray bars) than in fed controls (white bars; *p* < 0.001, One-way ANOVA). Like in **(A)** all genotypes display significantly lower Dilp levels after 24 h starvation (not indicated in Figure). **(C)** *Dilp5* transcript levels also increased significantly in fed *Dsk* deficient flies (*Dilp2/Dsk*-RNAi and *Dsk/Dsk*-RNAi) compared to controls (*p* < 0.001, One-way ANOVA). Like in **(A)** all genotypes display significantly lower Dilp levels after 24 h starvation (not indicated in Figure).

The levels of *Dilp* transcripts have been shown to be differentially affected when flies were subjected to different diets or other manipulations of IPC activity (Hwangbo et al., 2004; Broughton et al., 2008, 2010; Buch et al., 2008; Min et al., 2008; Karpac et al., 2009; Grönke et al., 2010; Slack et al., 2010; Birse et al., 2011). We analyzed *Dilp* transcript levels in fly brains in response to *Dsk*-depletion in IPCs or DPCs to determine if *Dsk*-levels have an effect on *Dilp* levels in fed or starved flies. Analysis by qPCR showed that the levels of *Dilp2*, *3*, and *5* increase when *Dsk* is knocked down in either IPCs or DPCs in flies that are fed *ad libitum* (*p* < 0.001 for all three *Dilps*, One-way ANOVA, Tukey post test; Figures 6A–C). In flies exposed to 24 h starvation, the *Dilp2*, *3*, and *5* levels decrease in both *Dsk*-knockdown flies and all controls (*p* < 0.01, One-way ANOVA; Figures 6A–C). Note that the *Dilp2* levels in flies starved for 24 after *Dsk*-knockdown are not significantly different from those seen in starved controls (n.s., Two-way ANOVA). Diminution of *Dsk* thus affects *Dilp* transcripts only in normally fed flies where the levels of *Dilp2*, *3*, and *5* increase to about 1.5- to 2-fold the levels seen in the controls. Interestingly, it is sufficient to knockdown *Dsk* in IPCs to obtain an increase in *Dilp* levels.

**Figure 7:**
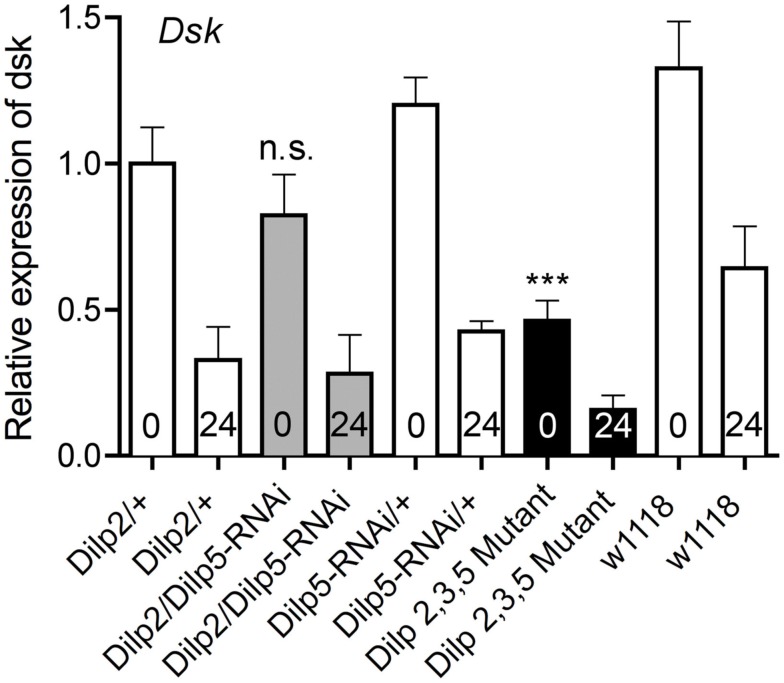
**Relative expression levels of *Dsk* transcripts in *Dilp* deficient files**. The *Dsk* transcript levels decreased significantly in normally fed *Dilp2,3,5* mutants (black bar) compared to controls (white bars; ***p < 0,001, One-way ANOVA, *n* = 10 for each genotype, assays run in triplicate, experiments in triplicate). Even though *Dsk*-levels drop significantly in all genotypes tested during starvation (*p* < 0,001, One-way ANOVA), the levels of *Dsk* mRNA after 24 h of starvation are lower in *Dilp2,3,5* mutants (black bar, 24) than in controls (white bars 24; *p* < 0,01, One-way ANOVA). Silencing only *Dilp5* in the IPCs with RNAi (*Dilp2/Dilp5*-RNAi; gray bars) did not affect *Dsk* transcript levels under normal fed conditions, nor is the drop in *Dsk*-levels significantly different from that seen in controls during starvation (n.s, Two-way ANOVA).

Since the IPCs express *Dilp2*, *3*, and *5* we utilized a *Dilp2,3*,5 mutant (Grönke et al., 2010) to knockdown insulin levels in the IPCs and measured the levels of *Dsk* transcript in fed and starved flies. In addition, we tested the effects of *Dilp5*-RNAi in IPCs on *Dsk* transcription levels. The *Dilp5*-RNAi was chosen because *Dilp5* levels were shown to be sensitive to starvation in this and other reports (Broughton et al., 2008; Min et al., 2008). Normally fed flies with mutated *Dilp2,3*,*5* display lower levels of *Dsk* than controls (Figure 7; *p* < 0.001, One-way ANOVA). After 24 h starvation, the levels of *Dsk* transcript decreased in all genotypes tested, including controls (Figure 7). This is as predicted since there is no need for a satiety signal during starvation. Knocking down *Dilp5* only in the IPCs did not significantly alter *Dsk*-levels in fed or starved flies compared to controls (n.s., One-way ANOVA, Tukey post test; Figure 7).

Taken together, the qPCR analysis above suggests a correlation between the *Dilp* levels and the *Dsk*-levels. The *Dilp* levels increase in *Dsk* deficient flies and *Dsk* transcript levels diminish in *Dilp2,3,5* deficient flies. A possible explanation for these results is a feedback where DSKs act on the IPCs either directly or via other cells to regulate *Dilp* transcription. Probably the increased *Dilp* levels in fed flies with reduced *Dsk* (and diminished satiety signaling) are caused by increased food intake leading to a demand for higher levels of circulating DILPs to reallocate carbohydrates. Furthermore, it is sufficient to diminish *Dsk* in IPCs to obtain an effect on *Dilp* levels. Similarly, the DILPs are likely to regulate *Dsk* transcription directly or indirectly via signaling from the fat body.

### Resistance to starvation

It is known that decreased DILP signaling leads to increased resistance to starvation and we wanted to determine whether altering the *Dsk*-levels affects survival in food deprived flies. We thus tested survival of *Dsk*-RNAi flies exposed to starvation by feeding aqueous agarose.

**Figure 8:**
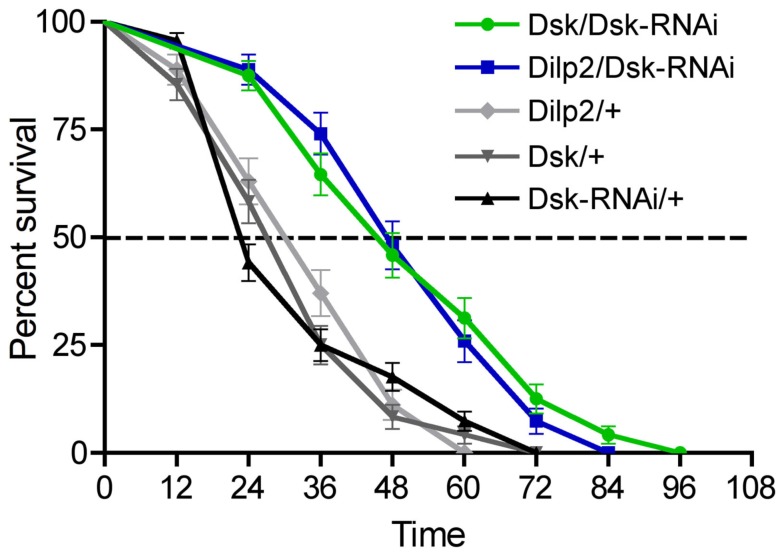
**Drosulfakinin and *Drosophila* insulin-like peptide deficient flies are more resistant to starvation**. We tested the effect of *Dsk*-knockdown in the IPCs (*Dilp2*-Gal4/*Dsk*-RNAi) and DPCs *Dsk/Dsk*-RNAi on survival at starvation. Using both Gal4 lines we obtained an extension of life span of the starved flies (*p* < 0.01 and *p* < 0.001, *n* = 70–95 for each genotype, run in triplicate, Log-rank test), compared to the controls Dilp2/+, Dsk/+, and Dsk-RNAi/+. The extension of lifespan seen here could be caused by the DSK and DILP deficient flies feeding more prior to the starvation experiment and thus having larger energy stores.

When the flies were deficient in *Dsk*, either by driving *Dsk*-RNAi in IPCs or DPCs (Figure 8) they displayed longer life spans at starvation than controls (*p* < 0.001 for both genotypes compared to controls, Log-rank test). It was shown earlier that partial ablation of the IPCs by expressing UAS-*rpr* produced the same phenotype (Broughton et al., 2005). However, the finding that *Dsk*-knockdown by RNAi in the IPCs (and DPCs) extends life span at starvation is a novel finding. This suggests that IPCs influence resistance to starvation not only by release of DILPs, but that also DSKs may have an impact on this resistance. Alternatively, the extension of life span is caused by the DSK-depleted flies feeding more vigorously before the start of the starvation experiment and therefore having larger energy stores.

## Discussion

Not only the structural resemblance between insect sulfakinins and mammalian CCK and their receptors is striking, but also the finding that sulfakinins act as satiety signals in both groups (Nichols et al., 1988; Wei et al., 2000; Maestro et al., 2001; Kubiak et al., 2002; Wu et al., 2003; Downer et al., 2007; Dockray, 2009). We show here that the *Drosophila* sulfakinins, DSKs, are important in regulating food intake and more specifically that DSKs in a set of brain neurosecretory cells (IPCs) that also produce insulin are sufficient for signaling satiety. Other studies made in locust, cricket, cockroach, and blowfly have employed various techniques to demonstrate that sulfakinins regulate feeding and satiety (Wei et al., 2000; Maestro et al., 2001; Downer et al., 2007; Meyering-Vos and Muller, 2007). For instance in the blowfly, *Phormia regina*, injected sulfakinins induce satiety for carbohydrate but not protein intake (Downer et al., 2007). We used targeted genetic interference with *Dsk*-levels or membrane activity in the specific neurons producing DSKs, the DPCs, and tested the effects on several aspects of feeding. Flies with diminished *Dsk* or activity in DPCs feed more vigorously than controls and are less selective in food quality. Most interestingly, it is sufficient to diminish DSK signaling from the IPCs to affect feeding. Our experiments suggest that the DILPs and DSKs released from the IPCs act in concert to regulate feeding and metabolism and also that the peptides display mutual feedback regulation of peptide gene transcription in these cells.

While co-localization of DSK and DILPs in brain IPCs was already known in *Drosophila* larvae (Park et al., 2008), we show here that the peptides are also co-localized in IPCs of the adult fruitfly. Most, but not all, of the IPCs produce DSKs at both developmental stages. It is not clear what the targets of DSK released from the IPCs are. Central neurons of the brain that regulate feeding is one possibility; peripheral targets associated with the alimentary canal or feeding apparatus is another. The cellular expression of the two DSK receptors (CG6857 and CG6881; Kubiak et al., 2002) is not known and consultation of the FlyAtlas^1^ (Chintapalli et al., 2007) and FlyBase^2^ reveals very low transcript levels for both receptors, with the highest for CG6857 in the brain, salivary gland, and fat body. Interestingly, the crop, crop duct, the proventriculus, and anterior midgut are supplied by axon terminations from the IPCs (Cao and Brown, 2001; Cognigni et al., 2011) and thus these could be targeted by DSKs. It may be that muscle contractions are regulated by DSK released directly onto these parts of the intestine, similar to CCK in mammals (Liddle et al., 1986; Dockray, 2009; Moran and Dailey, 2009). It is known that sulfakinins stimulate muscle contractions in insects (Nachman et al., 1986a; Melcher and Pankratz, 2005), but no specific action on the crop and proventriculus has been demonstrated (see Downer et al., 2007). However the peptide DSK-0 that is also encoded on the *Dsk* gene of *Drosophila* has been shown to induce contractions in the crop when applied at higher concentrations, indicative of non-hormonal action (Palmer et al., 2007). The sequence of DSK-0 is very different from DSK-1 and 2, and the peptide not likely to activate the two known DSK receptors.

Drosulfakinins are expressed in the IPCs, as well as other cells in the adult brain such as four large median posterior cells and some smaller cells in the protocerebrum and a few in the subesophageal ganglion (see also Nichols and Lim, 1996; Nichols et al., 1997a). Thus the *Dsk*-Gal4 driver that identifies all the DPCs includes the IPCs. By using a driver uniquely expressed in the IPCs (*Dilp2*-Gal4), we could distinguish the role of DSKs in the IPCs from that of global DSKs by expressing *Dsk*-RNAi. Significantly, when driving *Dsk*-RNAi in IPCs only, all of the phenotypes observed, including deregulated feeding, were the same as when driving *Dsk*-RNAi in all DPCs. This suggests that DSKs have an effect on regulation of food intake and food choice in *Drosophila* that indicates that the peptides signal satiety and also that the DSK released from the IPCs is sufficient for this signaling.

In our experiments, DSK does not only affect food intake, but also the food choice of larvae and flies. That the DSK deficient animals are less discriminating with food quality could depend on defective olfaction or taste, or that they are hungry enough to eat also noxious food. Nichols et al. (2008) showed that DSK has an effect on odor preference in *Drosophila* larvae. However, we observed the same alteration in food choice in flies with DSK reduced only in the IPCs, which probably excludes that taste or olfaction is deficient due to direct DSK modulation of these sensory systems. It is more likely that the DSK deficient flies or larvae do not receive satiety signals and thus remain in the non-preferred food and feed rather than to venture out to find another better food source. Defective food choice behavior has been seen in flies with impaired *hugin* and NPF signaling (Wu et al., 2003, 2005a,b; Melcher and Pankratz, 2005). In the case of NPF this altered behavior has been associated with insulin signaling; neurons expressing the NPF receptor are regulated by DILPs (Wu et al., 2005b; Lingo et al., 2007). Also the neuropeptides sNPF, leucokinin, and Allatostatin A are known to regulate food intake in *Drosophila* (Lee et al., 2004, 2008; Al-Anzi et al., 2010; Hergarden et al., 2012). Thus, the control of feeding behavior in *Drosophila* employs several peptides/peptide hormones, like in mammals (McMinn et al., 2000; Melcher et al., 2007; Moran, 2009). Our data adds DSKs to the set of different neuropeptides that regulates feeding and maybe indirectly metabolism in *Drosophila*.

We measured the *Dsk* transcript levels in flies mutant for *Dilp 2, 3*, and *5* and found a significant decrease in *Dsk*-levels under normal feeding conditions. This could either be caused by a direct feedback regulation of *Dsk* transcription by DILPs that is defective in the mutant, or by indirect feedback regulation via the fat body. For instance it is possible that the decreased levels of *Dsk* transcript observed in the *Dilp 2, 3*, and *5* mutant is due to a diminished ability for the fly to store energy because of the low circulating insulin levels and decreased signaling to the fat body. This might induce a perceived need for consumption of more energy, and thus a diminished need for a satiety signal, DSK. Thus the fat body may send a signal back to the brain IPCs leading to decreased *Dsk* transcription.

Our study shows that *Dilp 2, 3*, and *5* transcript levels are increased by diminishment of DSK-levels under normal feeding conditions. However, at starvation *Dilp* levels normally decrease and the *Dsk*-knockdown does not seem to affect this drop. This is consistent with the idea that DSK signaling does not occur during starvation as there is no need for a satiety signal. The increased *Dilp* levels in fed flies with reduced *Dsk* are probably caused by increased food intake leading to a demand for higher levels of circulating DILPs to reallocate carbohydrates.

Knockdown of different components of the insulin pathway results in an extension of life span at starvation (Clancy et al., 2001; Tatar et al., 2001; Giannakou et al., 2004; Hwangbo et al., 2004; Grandison et al., 2009; Enell et al., 2010). The increased survival observed when *Dsk* is knocked down indicates that DSK signaling might affect resistance to starvation, at least indirectly. However, it is possible that *Dsk*-knockdown flies feed more before the onset of the starvation experiment and thus have larger energy stores. It is thus not clear how DSK and DILP signaling acts in a coordinated fashion during starvation, or how DSKs might affect starvation resistance. Possibly also the DSKs act on the fat body, since at least one of the DSK receptors seem to be expressed in this tissue (reported in FlyAtlas)^3^.

In summary the *Drosophila* IPCs co-express DSKs and three DILPs, and the release of these peptides is likely to be coordinated. Thus, for any activation of the IPCs a cocktail of the two types of peptides is released into the circulation and it is likely that the action at different (or the same) targets is coordinated and orchestrates behavioral and metabolic events. At least after feeding the effect of the cocktail seems to be to induce satiety and a halted feeding at the same time as carbohydrate and lipid metabolism is altered to reallocate energy in the fly. This report is the first to identify a clear action of DSKs in regulation of feeding and satiety in *Drosophila* and also demonstrates mutual peptidergic feedback regulations of *Dilp* and *Dsk* gene transcription. Analysis of the distribution of the receptors for DSKs and DILPs would be helpful in the future to determine sites of peptide hormone action and to understand how the two types of signal could converge and possibly interact to regulate behavior and metabolism.

## Conflict of Interest Statement

The authors declare that the research was conducted in the absence of any commercial or financial relationships that could be construed as a potential conflict of interest.

## Footnotes

^1^http://www.flyatlas.org/

^2^http://flybase.org/

^3^http://www.flyatlas.org/
